# Self-rated health as a mediator between physical health conditions and depressive symptoms in older Chinese and Korean Americans

**DOI:** 10.1371/journal.pone.0245136

**Published:** 2021-01-08

**Authors:** Yuri Jang, Hyunwoo Yoon, Mengting Li, Nan Sook Park, David A. Chiriboga, Bei Wu, XinQi Dong, Miyong T. Kim

**Affiliations:** 1 Edward R. Roybal Institute on Aging, Suzanne Dworak-Peck School of Social Work, University of Southern California, Los Angeles, California, United States of America; 2 School of Social Work, Portland State University, Portland, Oregon, United States of America; 3 Institute for Health, Health Care Policy and Aging Research, Rutgers, The State University of New Jersey, New Brunswick, New Jersey, United States of America; 4 School of Nursing, Rutgers, The State University of New Jersey, New Brunswick, New Jersey, United States of America; 5 School of Social Work, University of South Florida, Tampa, Florida, United States of America; 6 Department of Child and Family Studies, University of South Florida, Tampa, Florida, United States of America; 7 Rory Meyers College of Nursing, New York University, New York, New York, United States of America; 8 School of Nursing, University of Texas at Austin, Austin, Texas, United States of America; Catholic University of Korea College of Medicine, REPUBLIC OF KOREA

## Abstract

In the present study, we examined self-rated health as a mediator between physical health conditions (chronic diseases and functional disability) and depressive symptoms in older Chinese and Korean Americans. Using harmonized data (*N* = 5,063) from the Population Study of Chinese Elderly (PINE) and the Study of Older Korean Americans (SOKA), we tested direct and indirect effect models. In both groups, chronic diseases and functional disability were closely associated with negative ratings of health and symptoms of depression. Analyses with the PROCESS macro showed that the effect of chronic diseases and functional disability on depressive symptoms was mediated by self-rated health in both groups; the indirect effect was greater in the Korean American sample than in the Chinese American sample. These findings contribute to the understanding of the psychological mechanisms that underlie the mind–body connection and highlight the potential importance of subjective health assessment as a useful tool for health promotion.

## Introduction

Over the past few decades, an extensive literature has demonstrated the linkage between physical health conditions and mental well-being in the later years of life. Deterioration of physical health is often accompanied by diminished mental well-being, and this mind–body connection reflects the multidimensional nature of health observed in many groups of older adults [1–3], including older Chinese and Korean Americans [4–6]. Although race and ethnicity are critical social determinants of health that often serve as sources of health disparities [7, 8], common mechanisms may underlie the link between physical and mental health across racial and ethnic groups. Identification of such mechanisms should not only improve our understanding of the health and well-being of diverse populations but also guide our effort to develop strategic health interventions.

One plausible mechanism in the relationship between physical health and mental well-being may involve self-rated health, because subjective appraisals of one’s own health may shape the mind–body connection [9–11]. The single question “How would you rate your overall health?” is a validated indicator of general health and well-being [12–16]. Yet given equivalent levels of physical health, a wide range of individual variations in perceiving and appraising health status has been demonstrated [9–11]. Self-rated health, because of its subjectivity and strong connection to well-being, thus seems to represent an intervening step between physical and mental health. For example, the presence of chronic diseases and/or functional disability may prime older individuals to harbor negative self-evaluations of health, which in turn may lead to increased symptoms of depression. Previous studies have suggested that health perceptions not only apply within the domains of physical and mental health but also may mediate between them [9–11]. This intervening mechanism of self-rated health should be robust across different racial and ethnic groups, because subjective perceptions of physical health may influence mental well-being regardless of race/ethnicity.

In the present study, we examine the mediating role of self-rated health in two large Asian subgroups: older Chinese and Korean Americans. Due in large part to language barriers, older Asian Americans are often underrepresented in national studies which are based on English-only surveys [17–20]. Also, Asian American subgroups are often combined into one group, with their ethnic variations rarely considered. Constructing Asian American samples that reflect ethnic and linguistic diversities at a national level remains a challenge [19, 20]. For this study, we used two large sets of data on older Chinese and Korean Americans: the Population Study of Chinese Elderly (PINE) [21] and the Study of Older Korean Americans (SOKA) [22]. Although these two studies differ in design and sampling methods, both studies are culturally and linguistically sensitive and offer a unique opportunity to examine similarities and differences between two of the largest subgroups of Asian Americans. Data harmonization, merging data from different studies, has been followed in several cross-racial/ethnic studies [23, 24]. Because of its contribution to scientific understanding in response to health disparities, data harmonization has been strongly endorsed, and guidelines with procedural steps have been suggested [24]. Given that Chinese and Korean languages are ranked 2nd and 4th among those in the US population with limited English proficiency (LEP) [25], the harmonization of the PINE and SOKA datasets offers ways to enhance access to these hard-to-reach LEP populations and increase our understanding of health in vulnerable populations.

In examining how physical health conditions (chronic diseases and functional disability), self-rated health, and depressive symptoms might be associated in older Chinese and Korean Americans, we hypothesized that, in both samples, (1) physical health conditions and self-rated health would have direct effects on depressive symptoms and (2) the effects of physical health conditions on depressive symptoms would be mediated by self-rated health. In addition, we explored the potential of the mediation model moderated by ethnicity. Although we anticipated that the mediation mechanism would be common across groups, we hypothesized that there would be ethnic differences in the magnitude of the indirect effects. The findings for the common mechanism should shed light on intervention strategies for integrated health management and health promotion for broad ranges of older populations.

## Materials and methods

### Data

Data from two independently designed studies, the PINE and the SOKA, were used for the present analysis. The PINE is a population-based longitudinal study of older Chinese Americans age 60 and above in the greater Chicago area, using community-based participatory research [18, 21]. Culturally appropriate recruitment strategies ensured adequate community participation. Face-to-face home interviews were conducted by trained bicultural and bilingual interviewers. Launched in 2011, four waves of data have been collected. In the present study, to make the PINE’s data collection period comparable to the SOKA’s, we used the fourth wave (*N* = 3,124), collected in 2017−2019. Detailed information on the PINE is available elsewhere [18, 21].

The SOKA is a multi-state survey of Korean immigrants age 60 and older. In an effort to increase the generalizability of findings, sites for the SOKA were selected from states with differing proportions of the entire Korean population: California, New York, Texas, Hawaii, and Florida. In each state, a primary metropolitan statistical area with a representative proportion of Korean Americans was selected: Los Angeles, New York, Austin, Honolulu, and Tampa. Combined, these sites present a continuum of Korean population densities. Community-based samples were recruited by a team of investigators who shared the target population’s language and culture. At each site, the project began with the compiling of a database of Korean-oriented resources, services, and amenities; this database facilitated the research team’s efforts for community engagement and guided the selection of locations for data collection. In developing these databases and in their use at each site, community advisors’ input was actively solicited. All surveys took place in 2017−2018. The survey questionnaire was in Korean, developed using back-translation and reconciliation. The questionnaire was designed to be self-administered, but trained interviewers were on site for respondents who needed assistance. A total of 2,176 individuals participated in the survey. Detailed information on the SOKA is available elsewhere [22].

The PINE and SOKA studies were approved by the institutional review boards at the educational institutions where the two studies’ principal investigators were affiliated. Written informed consent was obtained from all participants. For our comparative analysis, the two datasets were reduced to include only individuals age 60 and older who were without cognitive impairment (Mini-Mental State Examination score >10). For each participant, the percent of missing in the variables used in the present investigation was calculated, and those with more than 10% of data missing were excluded. These data management procedures left a total of 5,063 participants: 3,002 from PINE and 2,061 from SOKA. As missing cases were rare in the remaining data, pairwise deletion was used for each set of analyses.

### Measures

Guided by the National Institute on Aging’s guidelines for retrospective data harmonization [24], we processed PINE and SOKA for (1) identification of common variables and evaluation of those variables’ harmonization potential, (2) development of a common data format, and (3) integration and analysis of the harmonized data. Both datasets include information on several aspects of health and well-being. For this study, we selected comparable survey items from the two datasets and recoded responses to the items when necessary, as follows.

#### Chronic diseases

Participants in both studies were asked to indicate whether a medical professional had ever told them that they had specific chronic diseases or conditions, which were presented on a checklist. The two studies did not use identical checklists, so we selected six diseases that they did share: heart disease, stroke, cancer, diabetes, hypertension, and arthritis. Affirmative responses to individual items were summed for total counts.

#### Functional disability

Ten items for functional disability were from the basic and instrumental activities of daily living scales [26, 27], including eating, dressing, bathing, walking, transferring, grooming, toileting, managing money, using the telephone, and managing medications. Due to the difference in response formats (a 4-point scale in the PINE and a 3-point scale in the SOKA), responses were dichotomized (0 = *no help needed*, 1 = *in need of help*). Total scores could range from 0 (*no disability*) to 10 (*severe disability*). Cronbach’s alpha was high in both samples (0.85 for Chinese Americans, 0.87 for Korean Americans).

#### Self-rated health

Self-rated health was indicated by responses to the question “How would you rate your current health?” Due to difference in response formats (a 4-point scale in the PINE and a 5-point scale in the SOKA), responses were recoded as 1 = *excellent/very good*, 2 = *good*, 3 = *fair*, 4 = *poor*. For its potential role as a mediator, self-rated health was treated as a continuous variable.

#### Depressive symptoms

In the PINE, the Patient Health Questionnaire-9 (PHQ-9) [28] was used to index symptoms of depression. In the SOKA, the PHQ-2 [29], a short form of the PHQ-9, was used. We selected the two items that were common to both datasets for our analysis. These items pertained to the frequency of having “little interest or pleasure in doing things” and feeling “down, depressed or hopeless” over the past 2 weeks. Each item was scored on a 4-point scale ranging from 0 (*not at all*) to 3 (*nearly every day*). Total scores could range from 0 to 6, with higher scores indicating greater levels of depressive symptoms. Cronbach’s alpha was high in both samples (0.70 for Chinese Americans, 0.80 for Korean Americans).

#### Control variables

Demographic information included age (in years), gender (0 = *male*, 1 = *female*), marital status (0 = *married*, 1 = *not married*), education (0 = *≤high school graduation*, 1 = *>high school graduation*), and length of stay in the U.S. (years).

### Analytical strategy

After harmonizing the data, we compared the descriptive characteristics of the samples using *t* or chi-square tests. Bivariate correlations were performed to understand underlying associations among study variables. Group comparisons of correlation coefficients of major study variables were conducted using Fisher’s *r* to *z* transformation. We also conducted multivariate linear regression analyses of depressive symptoms in each group. The direct effects of chronic diseases and functional disability on depressive symptoms were tested, followed by entry of self-rated health. Using the PROCESS macro [30, 31], we examined the hypothesized mediation of self-rated health (i.e., indirect effects of chronic diseases/functional disability on depressive symptoms through self-rated health) and potential ethnic differences (i.e., moderating effects of ethnicity). The 95% confidence intervals for the effects were estimated using 5,000 bootstrap samples. Analyses were conducted after controlling for the effects of background variables (age, gender, marital status, education, length of stay in the U.S.). All analyses were performed using IBM SPSS Statistics 27 (IBM Corp., Armonk, NY).

## Results

### Characteristics of the sample

Table 1 summarizes the characteristics of the older Chinese and Korean American samples. Compared with the Chinese American sample, the Korean American sample was younger and included more women and those who were not married, with higher education and more years in the U.S. Both samples were comparable in number of chronic diseases. In both groups, the most prevalent condition was hypertension (56.4% in older Chinese Americans and 50.3% in older Korean Americans), followed by arthritis and diabetes (not shown in tabular format). Older Korean Americans had a higher level of functional disability, but their self-ratings of health were more positive. Older Korean Americans demonstrated a higher level of depressive symptoms than did older Chinese Americans. Reflecting the community-dwelling nonclinical nature of the sample, the distributions of scores for functional disability and depressive symptoms were skewed. To approximate a normal distribution, we conducted a log transformation, and the transformed scores were used in the statistical analyses.

**Table 1:** Comparisons of the sample characteristics.

	Older Chinese Americans (*n* = 3,002)		Older Korean Americans (*n* = 2,061)		*χ*^2^ (*t*)
	%	*M* ± *SD*	%	*M* ± *SD*	
Age		74.9 ± 7.99		73.2 ± 7.93	(7.39***)
Gender					
Male	39.0		33.2		19.0***
Female	61.0		66.8		
Marital status					
Married	67.8		60.8		26.4***
Not married	32.2		39.2		
Education					
≤high school graduation	76.8		60.3		158.3***
>high school graduation	23.1		39.7		
Years in the United States		22.8 ± 12.4		31.4 ± 12.1	(−24.4***)
Chronic disease		1.43 ± 1.10		1.39 ± 1.16	(1.36)
Functional disability		0.30 ± 1.05		0.37 ± 1.28	(−2.23*)
Self-rated health		2.68 ± 0.83		2.39 ± 0.86	(11.8***)
Depressive symptoms		0.25 ± 0.80		1.03 ± 1.54	(−23.3***)

* *p* < .05.

*** *p* < .001.

### Bivariate correlations among study variables

Correlations among study variables were examined in each sample, and the results were summarized in Table 2. Chronic diseases, functional disability, self-rated health, and depressive symptoms were significantly associated in older Chinese Americans (*r*s = .10 to .20, *p* < 0.001) and older Korean Americans (*r*s = .20 to .46, *p* < 0.001). Comparisons of correlation coefficients between the two groups showed that older Korean Americans had stronger correlations than did older Chinese Americans between chronic diseases and functional disability (*z* = −3.26, *p* < .01), chronic diseases and self-rated health (*z* = −10.3, *p* < .001), functional disability and self-rated health (*z* = −5.13, *p* < .001), chronic diseases and depressive symptoms (*z* = −2.16, *p* < .05), functional disability and depressive symptoms (*z* = −3.58, *p* < .001), and self-rated health and depressive symptoms (*z* = −7.62, *p* < .001).

**Table 2:** Correlations among main study variables in older Korean and Chinese Americans.

	1	2	3	4
1. Chronic disease				
Chinese American	──			
Korean American	──			
2. Functional disability				
Chinese American	.14***	──		
Korean American	.23***	──		
3. Self-rated health				
Chinese American	.20***	.14***	──	
Korean American	.46***	.28***	──	
4. Depressive symptoms				
Chinese American	.14***	.10***	.18***	──
Korean American	.20***	.20***	.38***	──

*** *p* < .001.

### Multivariate regression models of depressive symptoms

Table 3 includes regression models of depressive symptoms in older Chinese and Korean Americans. In both samples, the direct effects of physical health conditions (chronic diseases and functional disability) were significant when background characteristics were controlled. More chronic diseases and greater functional disability were significant predictors of depressive symptoms. The initial model accounted for 4% of the variance in older Chinese Americans and 9% in older Korean Americans. When self-rated health was included, the explained variance increased by 2% in older Chinese Americans and 7% in older Korean Americans. In both groups, poorer ratings of health were associated with higher levels of depressive symptoms. With the entry of self-rated health, the effects of chronic diseases and functional disability on depressive symptoms decreased in both groups, suggesting the potential mediating effect of self-rated health.

**Table 3:** Multivariate regression models of depressive symptoms.

	Standardized Regression Coefficient (β)			
	Older Chinese Americans		Older Korean Americans	
Physical health condition				
Chronic disease	.13***	.10***	.12***	.01
Functional disability	.09***	.07***	.15***	.11***
Potential mediator				
Self-rated health		.14***		.32***
Control variable				
Age	.01	.00	.00	−.02
Female	.03	.02	.01	−.01
Not married	.06**	.06**	.11***	.08***
>high school graduation	.04*	.06**	−.08**	−.03
Length of stay in the U.S.	−.08***	−.07***	−.02	.01
Δ*R*^2^	.04***	.02***	.09***	.07***
Overall *R*^2^	.04***	.06***	.09***	.16***

** *p* < .01.

*** *p* < .001

### The mediation effect of self-rated health and moderation by ethnicity

The PROCESS macro explored (a) the mediation model of self-rated health in the relationships between physical health conditions (chronic diseases, functional disability) and depressive symptoms and (b) potential moderation by ethnic group. In the models with chronic diseases, the indirect effect of self-rated health on depressive symptoms through self-rated health was significant for both older Chinese Americans (B [SE] = .002 [.000]) and older Korean Americans (B [SE] = .007 [.001]), as evidenced by the 95% bootstrap confidence interval (CI) for the indirect effect not containing zero in both groups ([.001, .003] in older Chinese Americans and [.005, .008] in older Korean Americans). A similar pattern was observed in the models with functional disability with a significant indirect effect for both older Chinese Americans (B [SE] = .01 [.003]) and older Korean Americans (B [SE] = .10 [.013]), with the 95% bootstrap CI not containing zero ([.009, .022] in older Chinese Americans and [.074, .127] in older Korean Americans).

Furthermore, the mediation models were moderated by ethnic group membership. In the model with chronic diseases, the difference between conditional indirect effects by ethnicity was significant (B [SE] = .005 [.001], 95% bootstrap CI = .003, .006). The magnitude of the indirect effect was greater in older Korean Americans (B [SE] = .007 [.001], 95% bootstrap CI = .005, .008) than in older Chinese Americans (B [SE] = .002 [.0003], 95% bootstrap CI = .001, .002). The significant modification by ethnicity in indirect effects was also observed in the model with functional disability (B [SE] = .08 [.01], 95% bootstrap CI = .06, .11), with the magnitude of the indirect effect being greater in older Korean Americans (B [SE] = .10 [.01], 95% bootstrap CI = .07, .13) than in older Chinese Americans (B [SE] = .02 [.003], 95% bootstrap CI = .01, .02). In other words, while the mediation mechanism of self-rated health was present in both groups, the magnitude of the indirect effect was greater in older Korean Americans than in older Chinese Americans.

## Discussion

Responding to the dearth of data on older Asian Americans and the persistent challenges in reaching out to them [17–20], we harmonized two sets of data from older Chinese and Korean Americans (PINE and SOKA), which were independently collected using culturally and linguistically sensitive approaches. With a combined sample size of 5,063, the harmonized data demonstrated similarities and differences in physical and mental health between older Chinese and Korean Americans and provided support for our hypothesized mediation model of self-rated health.

The first step was to compare basic characteristics of both samples. Chronic diseases and functional disability, both of which are common health risks and concerns in older populations, were used as indicators of physical health conditions. Older Chinese and Korean American samples were comparable in numbers and types of chronic diseases. On average, they had about 1.4 chronic diseases, with hypertension, arthritis, and diabetes the most common. However, as expected, there were significant differences on many indicators. The Korean sample showed higher scores on functional disability and depressive symptoms, and their self-rated health was more positive than that of the Chinese sample. The high levels of depressive symptoms observed in older Korean Americans are consistent with literature reporting mental health vulnerabilities of the Korean American population in general and its older members in particular [20, 32, 33].

We also found group differences in the level of association between independent and dependent variables. Although the measures of physical health conditions were closely associated with negative ratings of health and symptoms of depression in both groups, the strength of the relationships was consistently greater in the Korean American sample. The overall pattern was in accord with the widely appreciated mind–body connection, in which the presence of chronic diseases and functional disability makes older individuals prone to negative perceptions of health and symptoms of depression [1–3]. Multivariate analyses also demonstrated the direct effects of chronic diseases, functional disability, and self-rated health on depressive symptoms. In both groups, each variable made an independent contribution to explaining the variance of depressive symptoms, and the attenuated association between physical health conditions and depressive symptoms in the subsequent entry of self-rated health suggested a potential mechanism of mediation.

Further analyses with the PROCESS macro confirmed that self-rated health served as a common mediator across groups. Although the magnitude of the indirect effect varied, analyses of both older Chinese and Korean American samples supported the mediation model. The presence of chronic diseases and functional disability made older Chinese and Korean Americans perceive their overall health status in a negative manner, with an elevated level of depressive symptoms. Given the strong tendency of somatic manifestation of depressive symptoms in older Asian Americans [4–6], it is promising to target self-rated health for integrated health interventions. The stronger associations among health variables and the greater magnitude of the mediation effect observed in older Korean Americans could be attributed to nuanced ethnic differences in understanding and acceptance of physical and mental health and cultural beliefs regarding health, and further investigation of such speculation is warranted.

Adding support to previous findings for racially and ethnically diverse groups of older adults [9–11], our findings suggest that the intervening mechanism of subjective health perceptions in the relationship between physical and mental health is present regardless of race and ethnicity. Because positive self-rating of subjective health is strongly associated with health behaviors and well-being [12, 15, 16], these are important for developing health intervention programs for diverse populations. Along with disease prevention and health promotion efforts, it is also important to find ways to attenuate the negative mental health consequences of physical health conditions and subjective health perceptions could offer avenues for such interventions. Maintenance or enhancement of positive perceptions, optimistic attitudes toward health, and health-related control beliefs may serve as a bridge to optimized physical and mental health outcomes in older populations.

Some limitations to the present study should be noted. The foremost concern is the use of cross-sectional data in addressing a mediation model that is based on temporal assumptions. The potential for a reversed or reciprocal relationship among the constructs should not be ignored, because depressive symptoms could cause negative perceptions of health and deterioration in physical health. Although our findings suggest that the mediating role of self-rated health may be culture-free, the topic should be explored further with diverse groups of older adults longitudinally. It should also be noted that data harmonization allowed us to consolidate two existing independent datasets of under-studied populations often excluded in national surveys and to improve the generalizability of results. However, due to the discrepancies in measures across the studies, the scope of the current investigation was limited to the basic demographic and health-related variables. Although a significant amount of variance of depressive symptoms was explained by the selected measures, consideration of other variables such as family network, social support, and acculturation would enhance our understanding of social determinants of health and mental health. Despite these limitations, however, the present study contributes to research and practice by providing insights into a potential core mechanism underlying the mind–body connection in diverse older populations.
